# Prediction of clinical pregnancy in vitrified-warmed single blastocyst transfer cycles by pre-freeze morphology

**Published:** 2014-08

**Authors:** Huan Zhang, Ying Zhou, Yan Li, Yi Zheng, Shiquan Xiao, Yonggen Wu, Haiqing Wang, Xuefeng Huang

**Affiliations:** *Department of Reproductive Medical Center, The First Affiliated Hospital of Wenzhou Medical University, Wenzhou, China.*

**Keywords:** *In vitro fertilization*, *Innercellmass*, *Blastocyst*, *Vitrification*, *Morphology*

## Abstract

**Background:** The selection of blastocyst warmed for transfer is based on pre-freeze morphology in vitrified-warmed single blastocyst transfer cycles. But, it is controversial which parameter of blastocyst morphology most closely related to the clinical outcomes.

**Objective:** To estimate the effect of blastocoele expansion, trophectoderm (TE) morphology grade, and inner cell mass (ICM) morphology grade on clinical pregnancy in vitrified-warmed single blastocyst transfers.

**Materials and Methods: **There were 172 vitrified-warmed single blastocyst transfer cycles during the year 2012 included in this analysis. Comparison of clinical results between pregnancy and no pregnancy group based on patient and blastocyst morphology characteristics was done. Then stepwise logistic regression analysis was used to select the best morphological predictor for clinical pregnancy. Last, comparison of patient characteristics and clinical outcomes separated by the best independent morphological predictor was done.

**Results: **Comparison of clinical results between pregnancy and no pregnancy group and logistic regression showed the clinical pregnancy rate was affected by ICM. Comparison of patient characteristics separated by ICM grade, ICM grade A cycles got higher clinical pregnancy rate than ICM grade B cycles (54.3% vs. 35.0% respectively, p=0.037).

**Conclusion:** Blastocyst with good ICM morphology could increase clinical pregnancy rate in vitrified-warmed single blastocyst transfer cycles.

## Introduction

It has been reported that elective blastocyst transfer on day 5 could increase the implantation rate significantly compared to day 3 embryo transfer without increasing the risk of complications from multiple pregnancies (1). Nevertheless, it is still a challenge to select the best blastocyst from a group of sibling embryos. At present, morphology is an important tool used to predict embryo viability in vitro fertilization (IVF) cycles. Three morphological parameters, degree of blastocoele expansion, inner cell mass (ICM) and trophectoderm (TE) cells, are part of an established grading system, which are widely used for selecting blastocysts for embryo transfer. Several earlier attempts have been made to determine the separate impact of each parameter on implantation outcome, and even rank their importance (2, 3). 

Some investigators have shown that grade of expansion is the most important morphological predictor for assisted reproductive technology (ART) outcomes, some have been reported that the ICM grade has a most positive correlation with pregnancy rate, and other studies have demonstrated that the TE grade, but not the ICM grade, correlates with ART outcomes (4-8). So far, it is controversial which parameter of blastocyst morphology most closely related to the clinical outcomes. Furthermore, with the development of blastocyst cryopreservation technology, vitrified-warmed single blastocyst transfer has become an essential part of IVF/intra-cytoplasmic sperm injection (ICSI) treatment, increasing the cumulative pregnancy rate and reducing the incidence of multiple pregnancies. Although endometrial receptivity reportedly differs significantly between fresh and vitrified-warmed embryo transfer cycles, little articles have reported the effect of blastocyst morphology on the outcomes in vitrified-warmed transfer cycles (9). The predictive value of blastocysts morphology in vitrified-warmed cycles is not clear.

The purpose of the study was to evaluate the ability of morphologic assessment of the TE, ICM, and blastocyst expansion to predict pregnancy in vitrified-warmed single blastocyst transfer cycles.

## Materials and methods

This is a retrospective study performed between January 2012 and December 2012 in the First Affiliated Hospital of Wenzhou Medical University, Wenzhou, China. The treatments were conducted with patients following informed consent and according to the guidelines of the Ministry of Public Health of China (MPH). Inclusion criteria for this study were as follows: 1) the patient age was <45 years; 2) there was no evidence of an endocrinologic disorder (normal prolactin and thyroid-stimulating hormone levels); 3) the patient body mass index (BMI) was <35.0 kg/m^2^. All patients underwent controlled ovarian stimulation using a gonadotropin releasing hormone agonist (Diphereline, Ipsen, France), from either the mid-luteal phase of the preceding cycle in a long treatment protocol or second day of the cycle in a short treatment protocol, in combination with recombinant follicle-stimulating hormone (follitropin alfa, Serono, Switzerland) and/or human menopausal hormone. 

When one or more follicles reached a maximum diameter of 18 mm, human chorionic gonadotropin (HCG, Livzon, China) was administered. Transvaginal oocyte retrieval was performed 36 hours after hCG injection. 3-4 hours later, oocyte insemination was achieved with conventional in vitro fertilization or intracytoplasmic sperm injection as clinically indicated. After 16-18 hours, fertilization was confirmed by the presence of two pronuclei and two polar bodies. On the morning of day 3, two or three embryos were transferred and surplus embryos were transferred from cleavage medium to blastocyst medium (Vitrolife Medical, Sweden). Blastocyst quality on day 5 was assessed according to the criteria of Gardner and Schoolcraft (2). On Day 5, blastocysts of grade ≥2BC were considered available embryo and cryopreserved by vitrification.

Cryopreservation and warming of blastocysts were performed following the Cryotop methodology for human embryo vitrification described by Kuwayama (10). Equilibration, vitrification, warming, dilution, and washing solutions were provided in the Vit kit (Kitazato Biopharma, Shizuoka, Japan). Briefly, blastocysts were equilibrated in ES medium at room temperature for 8-12 minutes. Then they were placed into vitrification solution. After 1 minute in this solution, blastocysts were placed on the Cryotop strip then immediately submerged into liquid nitrogen. For warming, the Cryotop was taken out of liquid nitrogen and instantly placed TS at 37^o^C. After 1 min, blastocysts were placed in DS at room temperature for 3 min. Then 5 minutes washes were performed with WS1 at room temperature. Finally, blastocysts were washed with WS2 at 37^o^C for 5 minutes.

Hormone replacement therapy cycle will be performed for preparation of the endometrium. Between day 1 to day 5 of the cycle patients commence oral estradiol (Progynova, Bayer, Germany) 2 mg three times daily. After 11, 12 or 13 days an ultrasound was performed. If the endometrial thickness was ≥7 mm, micronized progesterone (Utrogestan, XianJu pharma, China) was added to the regime; if the endometrial thickness was less than 7 mm, the progynova dose was raised to 3 mg 3 times daily for 7 days, after a week the endometrium was checked once again, when the endometrial thickness was ≥7 mm utrogestan could be added. Then the single blastocyst was warmed and transferred 5 day later. Estradiol and progesterone supplementation were continued for the following 2 weeks up to the HCG test, if the test was positive, they were continued for another 2-3 weeks. Clinical pregnancy was identified by development of a gestational with fetal cardiac activity on ultrasound examination 4 weeks after blastocyst transferred. Miscarriage was deﬁned as an implanted embryo that failed to result in live birth.

**Statistical analysis**

All the statistical analyses were performed by using SPSS software (Statistical Package for the Social Sciences, version 19.0, SPSS Inc, Chicago, Illinois, USA). Student’s t-test was performed on continuous variables to determine differences in mean scores and standard deviation (SD). Categorical variables were analyzed using chi-square analysis. Stepwise logistic regression was used for selection of independent statistically significant predictors among the morphology variables and the confounders. Statistical significance was defined as p<0.05.

## Results

There were 172 vitrified-warmed single blastocyst transfer cycles during the year 2012 included in the analysis. All blastocysts transferred were frozen on day 5 in previous cycles. The pregnancy rate was 37.8%. Comparison of pregnancy and no pregnancy group in single blastocyst transfer cycles were performed based on blastocoele expansion grade, inner cell mass grade, trophectoderm grade and patient characteristics. As shown in table I, ICM grade and endometrial thickness were significantly associated with pregnancy. Stepwise logistic regression analysis of pregnancy demonstrated that the ICM grade was the only characteristic significantly associated with pregnancy (odds ratio [OR] 2.20, 95% confidence interval CI: 1.04-4.67, p=0.04). 

In this analysis, the adjusted variables included potential confounders such as blastocyst expansion, ICM, trophectoderm, female age at freezing, female age at transfer, BMI, basal FSH, duration of infertility, number of earlier cycles, endometrial thickness, primary infertility. Comparison of patient characteristics and clinical outcomes separated by ICM grade in vitrified-warmed single blastocyst transfer cycles is shown in table II. There were no differences in female age at freezing, female age at transfer, BMI, basal FSH, duration of infertility, number of earlier cycles, endometrial thickness, primary infertility, positive HCG and miscarriage rate, while ICM grade A transfer cycles had higher clinical pregnancy rate than ICM grade B transfer cycles (p=0.037).

**Table I:** Effects of patient and blastocyst morphology characteristics on pregnancy

		**Pregnancy**	**No pregnancy**	**p-value ** a
Transfers		67	105	
Female age at freezing (yr)		31.5 ± 4.0	31.3 ± 4.8	0.767
Female age at transfer (yr)		31.8 ± 4.0	31.6 ± 4.8	0.762
BMI (kg/m^2^)		21.2 ± 2.7	21.3 ± 2.9	0.845
Basal FSH (mIU/ml)		8.2 ± 2.6	8.3 ± 2.4	0.765
Duration of infertility (yr)		4.9 ± 2.4	4.7 ± 3.5	0.665
No of earlier cycles		1.4 ± 0.9	1.4 ± 1.1	0.778
Endometrial thickness (mm)		8.9 ± 1.3	8.5 ± 1.3	0.046 b
Primary infertility rate (%)		34.3	32.4	0.791
Expansion grade				
	4	19 (28.4%)	31 (29.5%)	0.382
	3	35 (52.2%)	45 (42.9%)	
	2	13 (19.4%)	29 (27.6%)	
ICM grade				
	A	19 (28.4%)	16 (15.2%)	0.037 b
	B	48 (71.6%)	89 (84.8%)	
TE grade				
	A	14 (20.9%)	22 (21.0%)	0.987
	B	44 (65.7%)	68 (64.8%)	
	C	9 (13.4%)	15 (14.3%)	

a Independent *t*-test and chi-square.

b significant differences (p<0.05) between pregnancy and no pregnancy group

**Table II:** Comparison of patient characteristics and clinical outcomes separated by ICM grade

	**ICM A**	**ICM B**
No of cycles	35	137
Female age at freezing (yr)	30.4 ± 4.0	31.6 ± 4.6
Female age at transfer (yr)	30.6 ± 4.0	31.9 ± 4.6
BMI (kg/m^2^)	20.9 ± 2.5	21.4 ± 2.9
Basal FSH (mIU/ml)	8.1 ± 2.8	8.3 ± 2.4
Duration of infertility (yr)	4.6 ± 2.4	4.9 ± 3.2
No of earlier cycles	1.34 ± 1.0	1.42 ± 1.0
Endometrial thickness (mm)	8.83 ± 1.2	8.56 ± 1.3
Primary infertility rate (%)	34.3	32.8
HCG positive rate (%)	54.3	43.1
Pregnancy rate (%)	54.3	35.0 a
Miscarriage rate (%)	15.8	16.7

a significant differences (p<0.05) between ICM A and ICM B group

## Discussion

The main finding of our study was that ICM grade was the most important morphological parameter for predicting clinical pregnancy in vitrified-warmed single blastocyst transfer cycles. By contrast, TE grade and blastocyst expansion were unrelated to clinical pregnancy. The advantage of the present paper was the use of single blastocyst transfers, making the assessment of each morphologic parameter with pregnancy possible, while the use of double-blastocyst transfers was a major weakness of former studies. And the use of logistic regression to control for cofounders further strengthens the conclusions. 

The blastocyst grading system of Schoolcraft et al has been used for more than a decade around the world. However, it was controversial which parameter of blastocyst morphology most closely related to the clinical outcomes. Richter *et al* reported that ICM was significantly related to the implantation rate (5). They found a higher implantation rate of Day 5 expanded blastocysts with ICMs of >4,500 square μm than those with smaller ICMs (55% vs. 31%). More recently, Kovacic *et al* reported ICM contributed more to blastocyst quality than TE, they found blastocysts with normal ICM and non-optimal TE in comparison with the opposite-normal TE and abnormal ICM had higher pregnancy rate (6). With the recent advent of embryo cryopreservation technology, vitrified-warmed transfer cycles have become an integral part of IVF, reducing the incidence of early onset OHSS (11, 12). However, there have been few reports on the relationship between pregnancy and single blastocyst evaluation in vitrified-warmed transfer cycles. In our single vitrified-warmed blastocyst transfer cycles study, ICM grade A cycles had higher pregnancy rate than ICM grade B cycles (54.3% vs. 35.0%, p=0.037). 

It was consistent with the studies of fresh blastocyst transfer cycles mentioned above. The relationship between ICM and pregnancy was not surpring, given that the ICM represents the group of cells destined to grow into the fetus. From studying mice, it has been established that isolated ICM from cavitating blastocyst can also implant in the utetus when transferred into surrogate mothers (13). In our study, the lack of association between TE grade and clinical pregnancy was surprising, given that it is the trophectoderm that forms the initial connection to the uterine wall and develops into the placenta and associated tissues supporting embryonic development. It may be explained by the following points. 

Firstly, it was a weakness that the blastocyst grading system of Schoolcraft *et al* only takes the number and conhesion of TE cells as the evaluation criteria, while several nuclei or vacuoles as well as necrotic foci cannot be assessed by cell-counting methods. It is possible that some as yet undetermined characteristic of the trophectoderm layer but no the number of TE cells could be indicative of blastocyst viability and implantation potential. Shapiro *et al* reported that they counted trophectoderm cells around an embryonic equator in one plane of focus and could not find any difference between the number of TE cells and clinical pregnancy (14). 

Secondly, it has established by studying mice, ICM can contribute the cells for TE, indicating the quality of blastocysts with lower TE grade can be compensating by ICM (15). Some studies found that blastocysts with higher expansion grades had higher chances of pregnancy in fresh blastocyst transfer cycles (4). But in our study, blastocoele expansion was not related to clinical pregnancy in vitrified-warmed single blastocyst transfers cycles. It may be explained that blastocysts with lower expansion grades had better intact survival after the processes of vitrified-warmed and compensated its lower implantation (16).

## Conclusion

In conclusion, these data suggest that selection of a blastocyst with good ICM morphology could increase pregnancy rate in vitrified-warmed single blastocyst transfer cycles.
